# Quorum sensing network in clinical strains of *A*. *baumannii*: AidA is a new quorum quenching enzyme

**DOI:** 10.1371/journal.pone.0174454

**Published:** 2017-03-22

**Authors:** María López, Celia Mayer, Laura Fernández-García, Lucía Blasco, Andrea Muras, Federico Martín Ruiz, German Bou, Ana Otero, María Tomás, on behalf of the GEIH-GEMARA (SEIMC)

**Affiliations:** 1 Department of Microbiology, Complejo Hospitalario Universitario A Coruña (CHUAC)-INIBIC, A Coruña, Spain; 2 Spanish Network for Research in Infectious Diseases (REIPI), Virgen Macarena, Seville, Spain; 3 Department of Microbiology, Faculty of Biology-CIBUS, University of Santiago de Compostela, Santiago de Compostela, Spain; 4 Biological Research Center (CIB-CSIC), Madrid, Spain; Tianjin University, CHINA

## Abstract

*Acinetobacter baumannii* is an important pathogen that causes nosocomial infections generally associated with high mortality and morbidity in Intensive Care Units (ICUs). Currently, little is known about the Quorum Sensing (QS)/Quorum Quenching (QQ) systems of this pathogen. We analyzed these mechanisms in seven clinical isolates of *A*. *baumannii*. Microarray analysis of one of these clinical isolates, Ab1 (*A*. *baumannii* ST-2_clon_2010), previously cultured in the presence of 3-oxo-C12-HSL (a QS signalling molecule) revealed a putative QQ enzyme (*α/ß hydrolase* gene, AidA). This QQ enzyme was present in all non-motile clinical isolates (67% of which were isolated from the respiratory tract) cultured in nutrient depleted LB medium. Interestingly, this gene was not located in the genome of the only motile clinical strain growing in this medium (*A*. *baumannii* strain Ab421_GEIH-2010 [Ab7], isolated from a blood sample). The AidA protein expressed in *E*. *coli* showed QQ activity. Finally, we observed downregulation of the AidA protein (QQ system attenuation) in the presence of H_2_O_2_ (ROS stress). In conclusion, most of the *A*. *baumannii* clinical strains were not surface motile (84%) and were of respiratory origin (67%). Only the *pilT* gene was involved in surface motility and related to the QS system. Finally, a new QQ enzyme (*α/ß hydrolase* gene, AidA protein) was detected in these strains.

## Introduction

Quorum Sensing (QS) is a general mechanism used by Gram-negative bacteria to regulate many biological processes, including virulence, competence, conjugation, resistance, motility and biofilm formation [1]. The production and detection of bacterial cell-cell signalling molecules by various species have been linked to the enhanced development of single and multi-species biofilms [2]. A variety of structurally different bacterial cell-cell signalling molecules have been shown to mediate cell-cell communication, including acyl homoserine lactones (AHLs) and autoinducer-2 molecules (AI-2). AHLs have been proposed to mediate intra-species bacterial communication; different species typically only recognize AHLs produced from closely related species [3]. On the other hand, AI-2 has been shown to mediate inter-species signalling [4]. The term AI-2 describes a family of inter-convertible molecules derived from the precursor molecule (4,5-dihydroxy-2,3-pentanedione, DPD) [5]. This precursor molecule is produced or detected by many Gram-positive and Gram-negative bacteria [6].

The QS system in *Acinetobacter* sp. has been described as homologous to the LuxR receptor (AbaR) and LuxI synthase (AbaI) proteins in *Vibrio fisheri* [7, 8]. Acyl homoserine lactones (AHLs) are classified on the basis of the length of the acyl chain as short- or long-chain molecules. Many strains of *Acinetobacter* (63%) produce more than one AHL (≥C10). Moreover, none of the AHL signals can be specifically assigned to any particular species of this genus [9]. In this pathogen, AHL molecules are autoinducers of the QS system involved in motility and biofilm production [10]. Secretion of quorum signals has been associated with multidrug efflux pumps [11, 12]. The AdeFGH efflux pump has recently been related to the synthesis and transport of autoinducers during biofilm formation regulated by the QS system in clinical strains [12]. Overexpression of the AdeABC efflux pump by deletion of the two-component regulatory system, AdeRS, has also been associated with biofilm formation and virulence phenotype in this pathogen [13]. However, little is known about the cascade of genes associated with various mechanisms controlled by the QS system in nosocomial pathogens such as *A*. *baumannii*. In an earlier study of the genes involved in QS activation in *A*. *baumannii* ATCC 17978, Clemmer *et al*. observed overexpression of an operon comprising the *A1S_0112* to *A1S_0118* genes [10].

The Quorum Quenching (QQ) mechanism can effectively interfere with any one of the key processes in QS, and this could potentially be exploited to quench QS and prevent microbial infections (inhibition of motility and biofilm formation) [14]. Naturally occurring QQ mechanisms act by blocking the key steps of QS, such as signal generation, signal accumulation and signal reception. Microorganisms exist in a multi-species, competitive environment and have developed many survival strategies to gain benefits and compete for space, nutrition and ecological niches. One of these, QS interruption, is straightforward because bacteria that produce QQ agents can inhibit the QS-regulated behaviour of competing species and therefore obtain benefits or avoid being killed. An AHL acylase, AmiE, has recently been identified in *Acinetobacter* sp. strain Ooi24 [15, 16]. However, this QQ mechanism is not well known in clinical isolates of *A*. *baumannii*.

In 2016, Vijayakumar *et al*. analyzed the nature of the clinical isolates of *A*. *baumannii* in relation to biofilm formation and motility [17]. These authors concluded that the least motile strains were those obtained from respiratory samples (the main origin of isolates of this pathogen). Interestingly, 67% of the non-motile clinical isolates in this study were of a respiratory nature. The oxygen-rich environment generates reactive oxygen species (ROS) (e.g. superoxide anion [O2^-^] and hydrogen peroxide [H_2_O_2_]), which can dramatically increase damage to cell structures in a process known as oxidative stress. To reduce the potential damage caused by these reactive intermediates, the bacteria possess ROS detoxifying enzymes (SOD and catalase proteins) [18]. Recent studies have suggested that the response to ROS is controlled by the QS system in *A*. *baumannii* [19, 20]. In all aerobically-grown microorganisms, the stress induced by ROS such as the superoxide anion radical (O_2_^-^) and hydrogen peroxide (H_2_O_2_) causes macromolecular damage [21].

In this study, we used microbiological and transcriptomic assays (microarrays and RT-PCR) to analyze the Quorum Sensing/Quenching systems in clinical strains of *Acinetobacter baumannii* in relation to surface motility. We also studied the involvement of these systems (QS/QQ) in the oxidative stress mechanism (ROS system).

## Material and methods

### Strains and susceptibility

Seven clinical strains shown by multilocus sequence typing (MLST) to have different allelic profiles or sequence types (STs) and different susceptibility to several antimicrobials were used in this study (Table 1). The mechanisms of resistance to several antimicrobials are shown in Table 2. The genomes of two isolates used in the study have already been sequenced: *Acinetobacter baumannii* ST-2_clon_2010 (Genbank acc.num. LJHB00000000) [22] and *Acinetobacter baumannii* strain Ab421_GEIH-2010 (Genbank acc.num CP014266) [23], Ab1 and Ab7 respectively. Both whole genome sequencing studies (WGS) are part of the GEIH-REIPI Spanish Multicenter *Acinetobacter baumannii* Study II 2000–2010, project PRJNA308422. The *abaR* and *abaI* genes were also sequenced in the present study.

**Table 1:** Clinical isolates of *A*. *baumannii* used in this study (molecular typing and antimicrobial resistance).

Strain	Molecular Typing	Antimicrobial resistance (MIC, mg/L)								
		IMIPENEM	MEROPENEM	TIGECYCLINE	GENTAMICIN	AMIKACIN	TOBRAMYCIN	CIPROFLOXACIN	COLISTIN	NETILMICIN
Ab1	ST-2	64	64	16	64	4	4	>64	≤0.5	16
Ab2	ST-186	64	64	≤0.25	≤0.5	4	0.5	16	≤0.5	≤0.5
Ab3	ST-52	16	16	≤0.25	32	<2	>64	64	≤0.5	>64
Ab4	ST-169	1	<0.5	0.5	<0.5	<2	1	<0.5	≤0.5	≤0.5
Ab5	ST-80	64	>64	32	64	128	1	64	≤0.5	32
Ab6	ST-181	64	>64	16	>64	128	<64	64	≤0.5	16
Ab7	ST-79	<0.5	1	8	>64	64	64	64	≤0.5	64

**Table 2:** Mechanisms of resistance to several antimicrobials in clinical isolates of *A*. *baumannii*.

Strain	ß-lactamase	Efflux pump (RND type)	Phenotype of antimicrobial resistance
Ab 1	OXA-24 (Plasmid harbouring AbKAB TA system)	Overexpression of AdeABC/AdeIJK	Carbapenems, aminoglycosides, quinolones and glycines
Ab 2	OXA-24 (Plasmid harbouring AbKAB TA system)	Not overexpression RND efflux pump	Carbapenems
Ab 3	OXA-58	Overexpression of AdeABC/AdeFGH	Carbapenems, aminoglycosides and quinolones
Ab 4	-	Not overexpression RND efflux pump	-
Ab 5	OXA-24 (Plasmid harbouring AbKAB TA system)	Overexpression of AdeABC/AdeIJK	Carbapenems, aminoglycosides, quinolones and glycines
Ab 6	OXA-24 (Plasmid harbouring AbKAB TA system)	Overexpression of AdeABC/AdeIJK	Carbapenems, aminoglycosides, quinolones and glycines
Ab 7	-	Absence of AdeABC efflux pump/ Overexpression AdeFGH	Aminoglycosides, quinolones and glycines

### Effect of culture conditions and quorum sensing inhibitors on motility

In view of the different responses shown by *A*. *baumannii* and *A*. *nosocomialis* to culture medium and Quorum Sensing inhibitors (Mayer *et al*., 2016, submitted), motility assays were performed in plates containing either Luria–Bertani (Normal LB) medium or modified LB-LN (nutrient depleted) [10]. The Normal LB medium contained (per litre) 10 g tryptone, 5 g yeast extract and 10 g NaCl per litre, while the modified LB contained 2 g tryptone, 1 g yeast extract and 5 g NaCl. Assays were carried out with 0.25% of Difco (Bacto^TM^ agar). Motility studies were carried out in the presence of QQ lactonase Aii20J enzyme, furanones and exogenous N-acyl homoserine lactone molecule (3-oxo-C12-HSL 10 uM).

The strains were inoculated in both normal LB and modified LB broth and incubated overnight at 37°C. An aliquot (1μl) of the culture was spotted in the centre of each well containing LB medium with agar and again incubated overnight at 37°C. Migration of the culture was then measured. The isolate was classified as non-motile when the average diameter of the zone of surface motility was <5 mm.

### Detection of the quorum quenching phenotype: AHL lactonase assay

All *A*. *baumannii* clinical strains were cultured overnight in 5 ml of modified LB broth. The culture was recovered by centrifugation at 3000 g for 10 min, resuspended, washed and finally resuspended in 5 ml of modified LB. Aliquots of the culture (1 ml) were supplemented with N-3-oxo-dodecanoyl-L-homoserine lactone (3-oxo-C12-HSL at a final concentration of 10 μM) and incubated at 37°C in a shaker for 6 h. Aliquots (50 μl) of the resulting supernatant were used to detect AHL degradation in a well diffusion assay in double agar plates, in which *Chromobacterium violaceum* CV026 was added to soft agar as a biosensor together with C6HSL (5 μM) to detect inhibition of violacein synthesis [24, 25]. An overnight culture of *E*.*coli* BL21(DE3)pET28-AidA strain was recovered and incubated in the presence of IPTG (Isopropyl β-D-1-thiogalactopyranoside) for 4 hours to induce expression of the *aidA* gene, and the medium was then supplemented with 3-oxo-C12-HSL. The diffusion assay was carried out as previously described [24, 25].

### Gene expression profiles

Gene expression studies were carried out by RT-PCR and microarray analysis. In both cases, the RNA samples were quantified in a NanoDrop ND-1000 Spectrophotometer (NanoDrop Technologies). The quality and integrity of the samples were analyzed in an Agilent 2100 Bioanalyzer, with RNA 6000 Nano reagents and RNA Nano Chips (Agilent Technologies). Only samples with an RNA integrity number (RIN) >8 were included.

**Fig 1:**
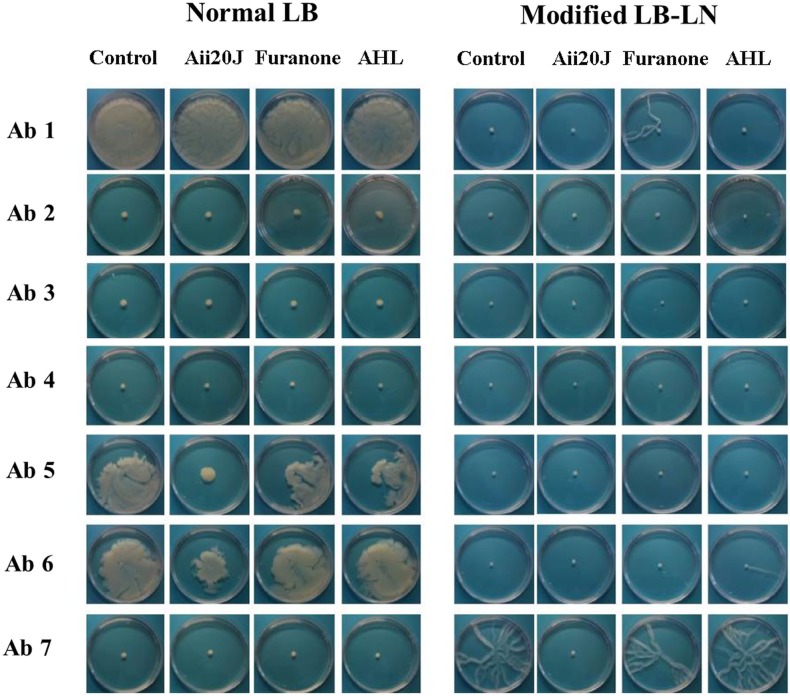
Surface motility of clinical strains of *Acinetobacter baumannii* on normal LB and modified LB (nutrient depleted). Normal LB contains 10g/L NaCL, 10g/L tryptone and 5g/L yeast extract and the modified LB contains 5g/L NaCL, 2g/L tryptone and 1g/L yeast extract. Inhibition of motility (in motile strains) was analyzed by the QQ enzyme (Aii20J) and molecules with this capacity (furanones and acyl homoserine, AHL).

For RT-PCR analysis of all clinical strains in both types of LB broth, the High Pure RNA Isolation Kit (Roche, Germany) was used to obtain Dnase-treated RNA from late log-phase cultures (OD = 0.4–0.6) in the presence of the QQ lactonase Aii20J at a final concentration of 20 μM [25] (Fig 1). The ROS experiments were carried out with the RNA extracts in the presence of H_2_O_2_ for 5 minutes and in the absence of H_2_O_2_. The primers and UPL probe (Universal Probe Library-Roche, Germany) used in RT-PCR analysis in all clinical *A*. *baumannii* strains are shown in Table 3. These were designed from QS genes of the operon comprising the *A1S-0112* to *A1S_0118* cluster of *A*. *baumannii* ATCC 17978 [10]. We also included the *abaR* and *abaI* genes (QS system) [26] as well as genes that encoded efflux pumps [11, 12]. In relation to the QQ system, we analyzed the *α/ß hydrolase* gene (AidA protein) by using several combinations of primers and probes with variations in the amino acid sequences (S1 Fig). Finally, we studied the ROS response associated with QS/QQ systems. The concentrations of the samples were adjusted to achieve efficiencies of 90%-110% (50 ng of RNA), and all experiments were performed in triplicate (i.e. three RNA extracts). Analysis of controls without reverse transcriptase confirmed the absence of DNA contamination. For each strain, the expression of all genes was normalized relative to the *rpoB* gene. The normalized expression of each gene of interest was calibrated relative to expression of the reference strains, which were assigned a value of 1.0 (mean relative expression: RE). The following comparisons were made relative to the reference strains: i) QS analysis. *A*. *baumannii* clinical isolates of interest due to their surface motility on both types of LB medium (Fig 1) *versus* strains in the presence of the QQ lactonase Aii20J enzyme (the same strains in the absence of the compounds were used as reference strains); and ii) ROS responses in relation to QS (AbaI protein) and QQ mechanisms (AidA protein) were compared in clinical isolates of *A*. *baumannii* in the presence of H_2_O_2_ (for 5 minutes) and in the absence of H_2_O_2_ (strains with no H_2_O_2_ pressure were used as reference strains, CONTROL).

**Table 3:** Primers and UPL probes used in this study.

PRIMERS and PROBES for RT-PCR STUDIES				
**Quorum Sensing**	**Primer Sequence (5´-3´)**		**Taqman Probes**	**Ref**
*A1S_0115*	Fow	TTGCCGGTTTGAAAAAGACT	11/CTTCCGC	This study
	Rev	TAAACGCACTTGGCACCATA		
*abaR*	Fow	ACCTCTTGTTTGGTCGAGTCA	96/ACAGGCAG	This study
	Rev	CGTGCTTCCTCCCAAAAAT		
*pilT*	Fow	CTTTGGTCTAGTGTGGTCATGC	102/TGGCTGAG	This study
	Rev	AAACAAAGTCGCGCAAATG		
*bfmS*	Fow	TGAAGGAGTCGCTCGACAA	38/GGAAGCAG	This study
	Rev	CAGATGCGTCAGAAATCCAAT		
*csuD*	Fow	CCTGAAAACCACCTAGTCGAA	60/TGGGGAAG	This study
	Rev	TTTTACTGGGTGACATTATTACCG		
*adeB*	Fow	CGAGTGGCACAACTAGCATC	61/CTGGGCAA	[27]
	Rev	CCTTGTCTTGGCTGCACTCT		
*adeG*	Fow	GTCCTGAAATGGTCGTTCGT	43/CTGCCCCA	[27]
	Rev	AGCTTCTGCTTGGCTAGATGA		
*adeJ*	Fow	CCTATTGCACAATATCCAACGA	119/TTGGTGGT	[27]
	Rev	AGGATAAGTCGCAGCAATCG		
*rpoB*	Fow	CGTGTATCTGCGCTTGG	131/CTGGTGGT	[27]
	Rev	CGTACTTCGAAGCCTGCAC		
*abaI*	Fow	CCGCTACAGGGTATTTGTTGAAT	6FAM-TGGATTCTCTGTCTTGAGCCACGACA-BBQ	This study
	Rev	GCAGGGAATAGGCATTCCATTG		
**Quorum Quenching**	**Primer Sequence (5´-3´)**		**Taqman Probes**	**Ref**
*aidA*	Fow	GGGAACTTCTTTCGGTGGAG	145/CAGCCACC	This study
	Rev	AACAGCAGCAAGTCGATTATCA		
	Fow	GGGACTTCTTTCGGTGGAG	145/ CAGCCACC	This study
	Rev	GCAGCAAGCCGGTTATCA		
	Fow	CCTAACCTTGCATTAGGGCTATTA	53/TGGCAGAG	This study
	Rev	CGGTAAACCACAGGTCGGTA		
**PRIMERS for SEQUENCING ANALYSIS**				
**Quorum Quenching**		**Primer Sequence (5´-3´)**		**Ref**
*aidA* gene (Putative lactonase)		AidA Fow	ATGGGTAAAAGTCTAAATAA	This study
		AidA Rev	CTTGACTGGAACGATG	
		AidA FowINT1	GCCTATGCACGTAGCC	This study
		AidA RevINT1	GGGGGCAACAGAGTCGG	
		AidA FowINT2	GCGAGATAGCCTGAAT	This study
		AidA FowINT2	GCCTATGCCCGTAGC	
**PRIMERS for CLONING the *aidA* gene in *E*.*coli* strain BL21 (DE3)**				
**Primer Sequence (5´-3´)**				
AidA pET Fow (***XhoI enzyme restriction site***)		GGAATTC***CATATG***GGTAAAAGTCTAAATAA		
AidA pET Rev *(****NdeI enzyme restriction site***)		GCG***GAGCTC***TTACTTGACTGGAACGATGCG		

**Ref**, References.

Overexpression of the genes was defined by RE values of ≥ 1.5. The differences in gene expression were analyzed using a Student’s t-test. Differences were considered significant at P<0.05 [27].

**Fig 2:**
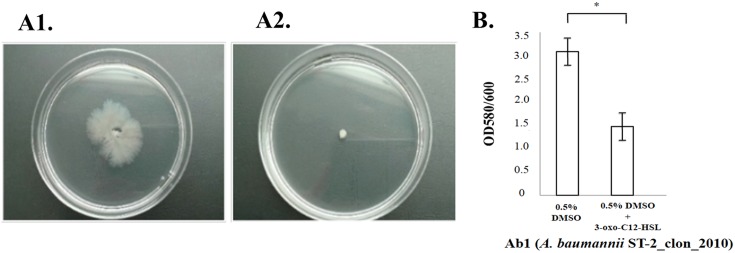
**The figure shows inhibition of motility (A2) and biofilm formation (B) in Ab1 in the presence of 3-oxo-C12-HSL (QQ activity).** As a negative control, motility and biofilm formation in Ab1 were studied in the presence of 0.3% dimethyl sulfoxide (DMSO), which is a solvent for 3-oxo-C12-HSL **(A1 and B)**.

In microarray studies (Fig 2), RNA was treated with 3-oxo-C12-HSL (significant signal molecule from QS of *P*.*aeruginosa*) at a final concentration of 10 μM [28] for analysis of clinical strain Ab1 (ST-2_clon_2010 isolate). Samples without enzymes and other compounds were used as negative controls. The arrays were designed using eArray (Agilent), on the basis of the Ab1 (ST-2_clon_2010 clinical strain genome) [22], and were carried out by Bioarray Diagnostico Genetico (Alicante, Spain). Labelling was carried out by two-colour microarray-based prokaryote analysis implemented with Fair Play III labeling, version 1.3 (Agilent). In this strain, the QS system was inhibited by 3-oxo-C12-HSL (100 μM) (i.e. inhibition of motility and biofilm formation, Fig 2). Four independent RNA extractions per condition (biological replicates) were used in each experiment. Statistical analysis was carried out using the Bioconductor tool of the RankProd software package for the R computing environment. A gene was considered overexpressed when the ratio of the treated to the untreated preparation was ≥1.5 at *P* <0.05.

### Biofilm formation

The biofilm assays were conducted following the procedure described by Álvarez-Fraga L *et al*. [29]. The strains were grown on LB medium for 18 h at 37°C and used to inoculate 5 mL of LB broth. Cultures were grown at 37°C with shaking. Overnight cultures were pelleted, washed and resuspended in 5 mL of modified LB-LN. A 1:100 dilution of each strain was incubated at 37°C for 48 h under static conditions. Growth of the culture was measured at OD_600_ to estimate total cell biomass. Biofilm formation was quantified by staining with crystal violet and solubilised with ethanol-acetone. The OD_580_/OD_600_ ratio was used to normalize the amount of biofilm formed to the total cell content of each sample tested, to overcome variations due to differences in bacterial growth under several experimental conditions. Eight independent replicates were considered. A student´s test was performed to evaluate the statistical significance of the observed differences between the strains considered.

### Sequencing of the *α/ß hydrolase* gene (AidA) in clinical strains of *A*. *baumannii*

The primers listed in Table 3 were used to sequence and amplify the gene that encoded the protein in all clinical strains isolated in this study. To confirm the absence of this gene, we analyzed the genome of Ab7 (*A*. *baumannii* strain Ab421_GEIH-2010 genome) [23]. Moreover, the nucleotide sequences of AbaR and AbaI proteins did not reveal any mutations of interest (data not shown).

### Expression of AidA protein in *E*.*coli* strains

DNA isolated from *Acinetobacter baumannii* strain ST-2_clon_2010 (Ab1) was used to clone the full-length AidA protein. The primers used to clone this gene in *E*.*coli* strains are shown in Table 3. These primers included an internal XhoI restriction site (bold), with a stop codon (underlined). The amplified PCR products were digested using NdeI and XhoI (NEB) and purified using a QIAquick PCR Purification Kit (QIAGEN). Finally, they were ligated with T4 DNA ligase (Fermentas) into a modified pET28-a plasmid (Novagen) which includes a human rhinovirus 3C protease cleavage site. Recombinant plasmids were transformed into competent *E*.*coli* DH5α cells (Novagen) for DNA production and purification (QIAprep Spin Miniprep Kit, QIAGEN). The integrity of both constructs was verified by sequencing. Finally, the plasmids were transformed into BL21(DE3) pLysS competent cells (Novagen) to yield the *E*.*coli* BL21(DE3)pET28-AidA construct.

## Results

### Surface motility in clinical strains of *A*. *baumannii*

The motility data for all clinical strains of *A*. *baumannii* are shown in Fig 1. Around 85% of the isolates did not exhibit surface motility on either medium tested. In those strains displaying surface motility, the lactonase Aii20J was the most effective in inducing QQ activity and thus inhibiting the motility (Ab 5 and Ab 6 on normal LB *versus* Ab 7 on modified LB-LN).

### Genes associated with surface motility (activation of the QS system)

We used RT-PCR to study the following QS genes in the clinical strains of *A*. *baumannii* of interest in relation to surface motility (shown in Fig 1): *A1S_115*, *abaR*, *pilT*, *bfmS*, *csuD*, *adeB*, *adeG* and *adeJ* [10–12].

We observed only one gene, *pilT*, associated with surface motility on both types of LB medium and involved in the QS system, as indicated by a decrease in expression (RE between 1.5 and 8 times higher) in the presence of the QQ lactonase Aii20J enzyme (Ab 5 on Normal LB and Ab 7 on modified LB-LN). The most strongly expressed gene was *A1S_115* in Ab3 (RE > 100 times higher). However, as clinical strain Ab3 did not display surface motility, this gene may be associated with another function.

Finally, in the Ab3 strain, overexpression of the genes that encoded the proteins AdeB (AdeABC) and AdeG (AdeFGH) decreased significantly in presence of the QQ lactonase Aii20J enzyme. In the other clinical strains, the differences were not statistically significant.

### Genes involved in inhibition of surface motility (activation of the QQ system)

Stacy *et al*. used non-native N-acyl homoserine lactones such as 3-oxo-C12-HSL to study attenuation of the QS system (QQ activity) [28]. We carried out microarray analysis of *A*. *baumannii* ST-2_clon_2010 (the genome of which has been sequenced) cultured with 3-oxo-C12-HSL, in order to analyze the expression profile of the genes involved in the QQ system in clinical strains of *A*. *baumannii* (the solvent used, dimethyl sulfoxide [DMSO] was included as a blank control). We also confirmed activation of the QQ system in this strain with 3-oxo-C12-HSL, by inhibition of surface motility and a significant decrease in biofilm formation (Table 4 and Fig 2).

**Table 4:** Gene expression in *A*. *baumannii* ST-2_clon_2010 (Ab1) revealed by microarray assays in the presence of 3-oxo-C12-HSL.

GenBank no. Sequences^a^	Gene name	Fold change	Function
GI:1056209154	Alpha/beta hydrolase	5.01	Quorum Quenching
GI:1056211401	Acyl-dehydrogenase (Acyl-CoA dehydrogenase)	4.83	AHLs synthesis
GI:1056209152	Short-chain dehydrogenase (3-oxoacyl-ACP reductase)	4.79	
GI:1056211410	Amp-binding enzyme (Acyl CoA synthase)	4.31	
GI:1056211399	Non-Ribosomal Peptide synthase	3.91	
	(Long chain fatty-acid CoA ligase)		
GI:1056209152	Kr domain protein (3-oxoacyl-ACP reductase)	3.27	
GI:1056211405	Acyl-homoserine-lactone synthase	3.18	
GI:1056211426	Phosphopantetheine attachment domain	2.72	
	(Beta-ketoacyl-ACP synthetase)		
GI:1056212294	Glutatione-S-transferase	2.56	Detoxification
GI:1056211398	Mmpl family protein (RND transporter)	1.89	Efflux pump
GI:1056212337	Outer membrane protein omp38	1.61	OmpA porin
GI:1056212369	Enterocidin EcnA/B family	1.51	Stress response
GI:1056211397	Porin	1.50	Porin

Only 13 genes were overexpressed in the presence of 3-oxo-C12-HSL in this *A*. *baumannii* strain (GEO database arrays GSE87009). The most strongly expressed gene (5.01) was a gene encoding an α/ß hydrolase enzyme, AidA (putative QQ ENZYME, GI:1056209154). Around 46.15% of the genes that were overexpressed were involved in the synthesis of the acyl-homoserine lactones (AHLs), including AHL synthase (GI:1056211405). Moreover, the results of the microarray analysis revealed overexpression of the genes coding for the following proteins: i) glutathione-s-transferase (DETOXIFICATION, GI:1056212294); ii) RND efflux pump (TRANSPORTER, GI:1056211398); iii) outer membrane protein-OmpA-Like (TRANSPORTER/VIRULENCE, GI:1056212337); iv) entericidin EcnA/B family (STRESS RESPONSE, GI:1056212369); and v) a new PORIN (GI:1056211397) (Table 4).

### Detection of the *aidA* gene in clinical isolates

To confirm the role of AidA as a new QQ enzyme, we studied the presence of this protein in the clinical strains of *A*. *baumannii* that did not display surface motility on modified LB-LN (Ab2, Ab3, Ab4, Ab5 and Ab6) and in the only strain displaying surface motility (Ab7). We found the α/ß hydrolase gene encoded an AidA protein (QQ enzyme) in all non-motile isolates (67% from respiratory tract) (Table 5). The AidA enzyme showed variable levels of amino acid in different clinical strains of *A*. *baumannii* (S1 Fig). Importantly, the α/ß hydrolase gene was not amplified in strain Ab7 (reference strain displaying surface motility). The absence of this gene was confirmed by sequencing the genome of strain Ab7 (which belongs to the PFGE-HUI-1 clone), which has recently been published as *A*. *baumannii* strain Ab421_GEIH-2010 [23]. Other characteristics of this isolate were that it did not have an AdeABC efflux pump and it did not harbour OXA 24 ß-lactamase in a resistance plasmid (Table 5).

**Table 5:** Detection of the *α/ß hydrolase* gene (AidA protein) in clinical isolates by PCR. Features of the isolates used in this study.

Strain	Quorum Sensing system	Surface Motility	Type of infection
	*α/ß hydrolase* gene, AidA protein: GI:1056209154^a^	(Modified LB-LN)	
Ab 1	+	No	Respiratory
Ab 2	+	No	Ulcer
Ab 3	+	No	Respiratory
Ab 4	+	No	Respiratory
Ab 5	+	No	Respiratory
Ab 6	+	No	Exudade
Ab 7	-	Yes	Blood

^**a**^ Genome *A*. *baumannii* ST-2_clon_2010.

**Fig 3:**
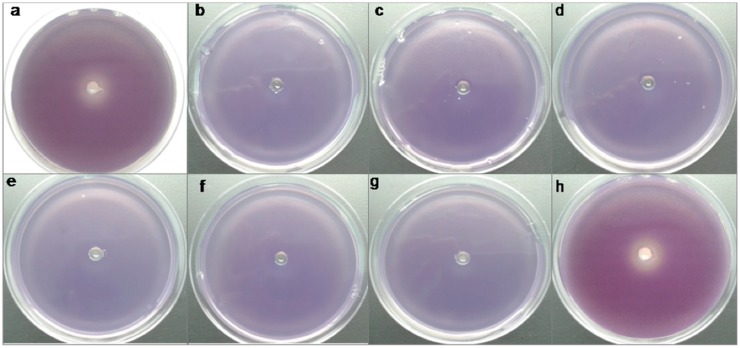
Diffusion assays carried out with the biosensor *Chromobacterium violaceum* CV026 to detect the presence of QQ activity in the clinical strains of *A*. *baumannii* under study. The presence of a halo indicates inhibition of violacein production by the presence of 3-oxo-C12-HSL and, therefore, the absence of QQ activity (a, h). The absence of a halo indicates QQ activity (b, c, d, e, f, g). a) Control; b) Ab1; c) ab2; d) Ab3; e) Ab4; f) Ab5; g) Ab6; h) Ab7.

Finally, in order to detect the presence of QQ activity in the *A*. *baumannii* strains used in this study, we conducted well diffusion assays with *Chromobacterium violaceum* CV026 as a biosensor. This bacterium produces violacein in the presence of short-chain AHLs such as C_6_HSL; however, the presence of long-chain AHLs inhibits violacein production [24]. In all strains except Ab7 (the reference strain exhibiting surface motility) and the control strain, no halo was detected in the plates, and therefore the 3OC12HSL with which the cultures were incubated was not present (Fig 3).

### Functional characterization of the AidA protein by overexpression in *E*.*coli* strains

**Fig 4:**
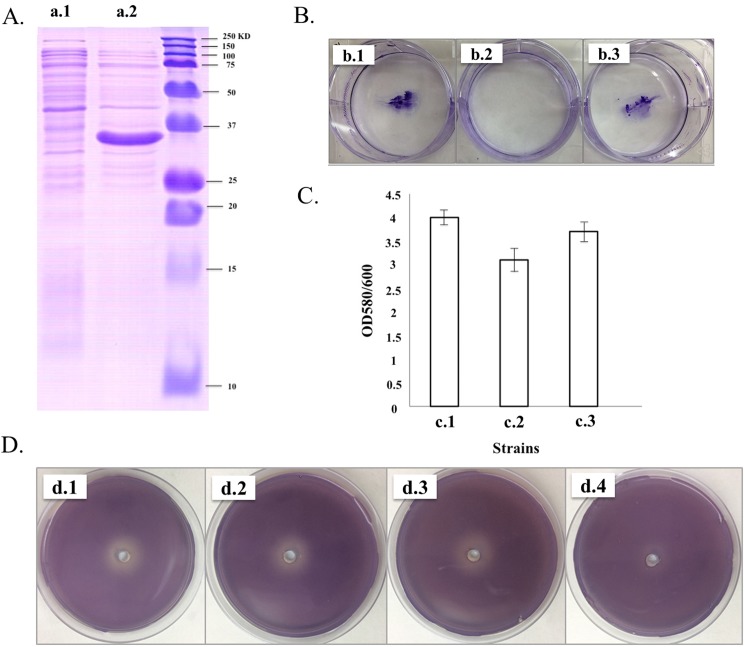
Studies with AidA protein overexpressed in *E*. *coli* BL21(DE3). **A.** SDS PAGE with expression of AidA protein in *E*. *coli* BL21(DE3): a.1. *E*.*coli* BL21(DE3)pET28 and a.2 *E*.*coli* BL21(DE3)pET28-AidA; **B.** Twitching motility: b.1 *E*.*coli* BL21(DE3), b.2 *E*.*coli* BL21(DE3)pET28-AidA and b.3 *E*.*coli* BL21(DE3)pET28; **C.** Quantification of biofilm formation by crystal violet staining: c.1 *E*.*coli* BL21(DE3), c.2 *E*.*coli* BL21(DE3)pET28-AidA and c.3 *E*.*coli* BL21(DE3)pET28. The differences were statistically significant (Student's t-test, P <0.05); **D.** Detection by plate diffusion assay of QQ activity after incubation in presence of 3OC12HSL: d.1. Control (LB); d.2. *E*. *coli* BL21(DE3); d.3. *E*.*coli* BL21(DE3)pET28; d.4. *E*.*coli* BL21(DE3)pET28-AidA.

We used *E*. *coli* BL21 (DE3) as a model to overexpress the AidA protein (pET28-AidA) (Fig 4A, line a.2). Three QS systems have been described in this model [26]:

1. Unknown (synthase), SdiA (receptor) and 3-oxo-C8-HSL (signal synthesized in others bacteria). This system has been associated with motility and acid resistance. [30].
2. LuxS (synthase), LsrB (receptor) and AI-2 (signal). Lsr operon expression (AI-2 uptake) [31].
3. Unknown (synthase), QseC (receptor) and AI-3 (signal synthesized in other bacteria). This system has been implicated in virulence, motility and biofilm formation [32].

We confirmed the quorum quenching activity of this AidA protein by twitching motility studies with these cells in the presence of IPTG (Fig 4B in which *E*.*coli* BL21 [DE3] pET28-AidA showed no motility) and quantification of bacterial biofilm (significant decrease in biofilm formation in *E*.*coli* BL21 [DE3] pET28-AidA, Fig 4C). The quorum quenching activity of this protein was also detected by diffusion assay in the presence of the biosensor *C*. *violaceum* strain CV026: no halo was produced by the supernatant of the strain BL21 [DE3] pET28-AidA incubated with 3-oxo-C12-HSL (Fig 4D).

### Relationship between QS/QQ systems and oxidative stress mechanism (ROS response)

We studied the involvement of QS/QQ systems in the ROS response by RT-PCR. In the presence of H_2_O_2_, expression of the *α/ß hydrolase* gene (QQ system) decreased significantly (P<0.05), while the *abaI* gene (QS system) was significantly overexpressed (P<0.05) (Table 6).

**Table 6:** Relative expression (RE) under normal conditions relative to the presence of H_2_O_2_ (ROS stress) in clinical strains of *A*. *baumannii* of genes involved in the QQ system (*α/ß hydrolase* gene, AidA protein) and the QS system (*abaI* gene, AHL synthase protein).

Strain	Quorum Quenching system		Quorum Sensing system	
	*α/ß hydrolase* gene, AidA protein: GI:1056209154^a^		*abaI* gene, AHL synthase protein: A1S_0109^b^	
	CONTROL (References)	H_2_O_2_	CONTROL (References)	H_2_O_2_
Ab 1	1	0.36	1	1.60
Ab 2	1	0.88	1	1.61
Ab 3	1	0.53	1	3.58
Ab 4	1	0.55	1	1.64
Ab 5	1	0.69	1	2.98
Ab 6	1	0.62	1	3.73
Ab 7	**-**	**-**	1	1.54

^**a.**^ Genome *A*. *baumannii* ST-2_clon_2010 (Ab1).

^b.^ Genome *A*. *baumannii* ATCC 17978.

Control: RNA extraction from clinical isolates under normal conditions; H_2_O_2:_ RNA extractions from clinical isolates after 5 minutes in the presence of H_2_O_2._

## Discussion

The QS system enables bacterial populations to live and proliferate in an environment with effective intercellular communication [33]. In clinical isolates of *A*. *baumannii*, little is known about the cascade of genes controlled by this system and associated with various mechanisms, including surface motility (phenotypic expression). In *Acinetobacter baumannii* ATCC 17978, the *A1S-0112* to *A1S_0118* operon has been associated with activation of the QS system, and the *pilT* gene has been related to motility [10]. In this study, only *pilT* expression (surface motility) was controlled by QS in the LB broth used: normal LB and modified (nutrient depleted) LB-LN.

Amino acid sequences and architecture of the QQ enzymes are diverse [34]. These enzymes have several biological roles: QS-signal clearing in *A*. *tumefaciens* [35, 36], recycling of QS signals (*Pseudomonas aeruginosa* model) in organisms that produce QS molecules [37, 38], detoxification and, finally, disturbance of QS signalling by an organism that does not produce QS signals, but may take advantage of QQ processes, such as the hosts of QS-emitting pathogens (bacterial competition) [39].

Several α/ß hydrolase enzymes have been described in different pathogens [1, 34, 40–42]. In this study, we identified in presence of 3-oxo-AHL, a new QQ enzyme (α/ß hydrolase gene, AidA) which was present in all strains of *A*. *baumannii* that did not exhibit surface motility. Moreover, the QQ activity from this protein (inhibition of the motility and biofilm) was confirmed by overexpression in *E*.*coli* (which does not produce AHLs). Interestingly, Weiland and collaborators described several QQ enzymes with hydrolytic activity against AHLs and AI-2 signals [43, 44]. Hence, this QQ enzyme (AidA protein) could contribute in bacterial competition, as it is capable of hydrolyzing the signalling molecules mediated between species.

Recent studies suggest that the ROS response is controlled by the QS system in *A*. *baumannii* [14, 15]. Several studies have suggested the involvement of the QQ mechanism under ROS response [45, 46]. Veal *et al*. [47] and other researchers [48] have suggested that members of the glutathione-S-transferase family of proteins are important for protecting cells from oxidative stress. The results of microarray assays showed overexpression of the *glutathione-s-transferase* and *α/ß hydrolase* (AidA protein) genes in the Ab1 clinical strain (*A*. *baumannii* ST-2_clon_2010) [22] in the presence of 3-oxo-C12-HSL[28]. Moreover, in *Deinococcus radiodurans*, which is known for its resistance to oxidative stress, the AHL level was “shielded” by QQ enzymes under non-stress conditions (normal conditions), whereas AHLs accumulated when *D*. *radiodurans* was exposed to oxidative stress [49]. In the aforementioned study, the synthetic form of the AHL enzyme (DsqI) was immediately induced on exposure to H_2_O_2_, while the expression of QQ enzymes began to increase after exposure to H_2_O_2_ for about half an hour. The QS system (DqsIR) in this pathogen mediated the adaptive strategy in response to oxidative stress (ROS response) [49]. In the present study, we confirmed the presence of the AidA protein (new QQ enzyme) in all non-motile clinical strains of *A*. *baumannii*. The twitching motility and biofilm studies with overexpression of this protein in *E*.*coli* BL21(DE3) confirmed its role as a quorum quenching enzyme.

Finally, the AidA protein was downregulated (QQ system attenuation) in the presence of H_2_O_2_ (ROS stress), unlike the AbaI protein, which was upregulated in clinical strains of *A*. *baumannii*.

In conclusion, we researched the Quorum Sensing/Quenching systems in clinical isolates of *A*. *baumannii*. Most of the strains were not surface motile (84%) and were of respiratory origin (67%). Only the *pilT* gene was involved in surface motility and the QS system in these strains. A new QQ enzyme (*α/ß hydrolase* gene, AidA protein) was detected by array analysis in the presence of the external signal 3-oxo-C12-HSL. All of the non-motile strains of *A*. *baumannii* had the AidA protein (QQ system activation). The function of this protein as a QQ enzyme was confirmed by its expression in *E*.*coli* BL21(DE3) strain that produces AI-2 signalling molecules. Finally, the findings confirmed that regulation of ROS stress (presence of H_2_O_2_) by the QS/QQ systems in clinical strains of *A*. *baumannii*.

### Nucleotide sequence accession number

The Ab ST-2_clon GEIH-2010 (Ab1) whole genome shotgun project has been deposited in the DDBJ/ENA/GenBank under accession number LJHB00000000. The version described in this paper is version LJHB01000000, which consists of sequences LJHB01000001 LJHB01000077. The genome sequence of Ab421 GEIH- 2010 strain (Ab7) has been deposited in GenBank under the accession number CP014266. Both WGS studies are part of the II Spanish Multicenter Study. GEIH-REIPI *Acinetobacter baumannii* 2000–2010 project (PRJNA308422).

## Supporting information

S1 Fig Amino acid sequences of the AidA protein (new QQ enzyme) isolated from clinical strain. (TIF)
