# Supplementary figures and images for: Quorum sensing network in clinical strains of A. baumannii: AidA is a new quorum quenching enzyme
Source: PLoS One. 2017 Mar 22;12(3):e0174454. doi: 10.1371/journal.pone.0174454 (PMC5362224; doi:10.1371/journal.pone.0174454)

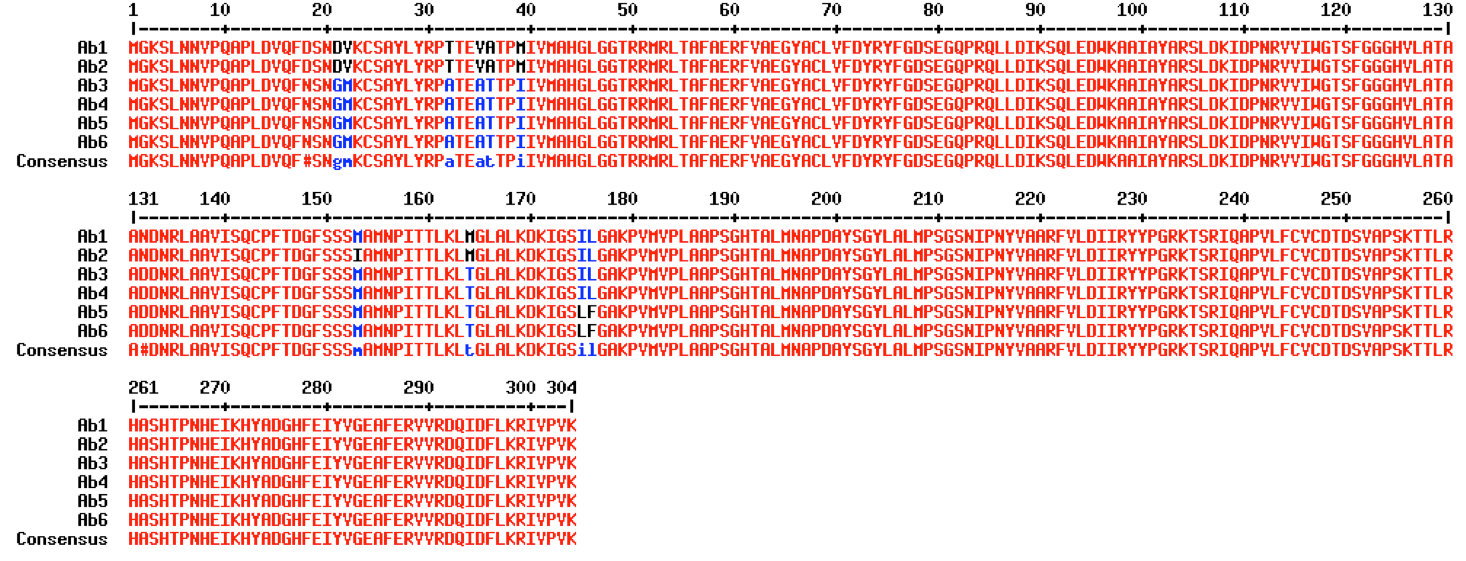

Supplement: S1 Fig — (TIF) [file pone.0174454.s001.tif]
